# Brexpiprazole augmentation in a clozapine-resistant young schizophrenic patient: a successful case report.

**DOI:** 10.1192/j.eurpsy.2023.2317

**Published:** 2023-07-19

**Authors:** V. Martiadis, F. Raffone, M. Russo

**Affiliations:** Department of Mental Health, ASL Napoli 1 Centro, Napoli, Italy

## Abstract

**Introduction:**

Although clozapine is considered the most effective drug for Treatment Resistant Schizophrenia (TRS), only 40% of patients treated will meet clinical response. Literature reviews and meta-analytic data offer no definite conclusions about the most effective clozapine augmentation strategies. Nevertheless, it has been suggested that the lack of evidence should not discourage clinicians from trying out new strategies in individual patients. Brexpiprazole is a novel 5-HT and DA modulator antipsychotic that exhibits partial agonism to D2/D3 and 5HT1A receptors, antagonism to 5HT2A and α-1B/2C receptors and represents a promising new drug in the pharmacotherapy of schizophrenia for both acute and maintenance treatment. In current literature there is no evidence of experiences in brexpiprazole augmentation for clozapine resistant patients.

**Objectives:**

This case report describes a successful clinical experience of brexpiprazole augmentation in a complicated case of clozapine resistant paranoid schizophrenia with consistent negative symptoms, the clinical evolution and metabolic improvement consequent to this therapy combination.

**Methods:**

In a 27 male TRS patient, sequentially treated with adequate doses of risperidone, cariprazine, aripiprazole and olanzapine monotherapy, prescribed for adequate time, clozapine treatment was started, with a gradual titration from 25 to 300 mg/day, without significant clinical response on both positive and negative symptoms. Successively was introduced fluoxetine, from 10 to 30 mg/day, with no relevant clinical improvement. After 2 months of pharmacological stabilization with clozapine and fluoxetine described dosages, brexpiprazole was introduced starting from 1 mg/day and rapidly increasing till 4 mg/day.

**Results:**

After 6 weeks of treatment, PANSS positive and negative subscales showed a significant decrease, respectively from 28 to 17 and from 42 to 20, while general psychopathology subscale decreased from 66 to 34. Negative sub-items with major improvement were those regarding blunted affect, emotional withdrawal, poor rapport, and passive/apathetic social withdrawal. Brexpiprazole augmentation also allowed to slowly decrease (in 6 months) clozapine dose till actual 150 mg/day, with a significant improvement in general tolerability and a slow decline in metabolic parameters (BMI from 36.5 to 30.3; fasting glucose from 112 to 92 mg/dL; total cholesterol from 248 to 182 mg/dL; total triglycerides from 392 to 198 mg/dL).

**Conclusions:**

In this case report brexpiprazole augmentation in a clozapine resistant young schizophrenic patient was an effective strategy with significant symptoms improvement, in particular in PANSS general psychopathology and PANSS negative subscales. The consequent clozapine dose reduction contribute to the slow decrease in metabolic parameters considered.

**Disclosure of Interest:**

None Declared

